# Laser Light Induced Transformation of Molybdenum Disulphide-Based Nanoplatelet Arrays

**DOI:** 10.1038/srep37514

**Published:** 2016-11-28

**Authors:** Arūnas Jagminas, Gediminas Niaura, Rokas Žalnėravičius, Romualdas Trusovas, Gediminas Račiukaitis, Vitalija Jasulaitiene

**Affiliations:** 1 State Research Institute Centre for Physical Sciences and Technology, Savanoriu Ave. 231, LT-02300 Vilnius, Lithuania; 2 Vilnius University, Faculty of Physics, Sauletekio Ave. 9, LT-10222 Vilnius, Lithuania

## Abstract

One-pot hydrothermal synthesis of MoS_2_ nanoplatelet arrays on various substrates is perhaps the most promising approach to fabricate efficient electrocatalysts for hydrogen evolution reaction. However, the main challenges in this synthesis remain the purity and crystallinity of MoS_2_. In this study, we show for the first time that irradiation of amorphous, defect-rich MoS_2_ nanoplatelets with a green nanosecond laser at a proper irradiation dose, ca ≤0.35 J cm^−2^, can significantly improve the crystallinity and purity of MoS_2_ nanoplatelets. The effect was confirmed by Raman spectroscopy investigations demonstrating a surprising intensity increase of the A^1^_g_ and 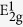 modes after the laser irradiation. Further increase of irradiation energy, however, resulted in the photocorrosion and destruction of MoS_2_ nanoplatelets. The variation of A^1^_g_ and 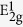 Raman mode intensities ratio depending on the green light irradiation dose was also presented and is discussed below.

Single and few-layered dichalcogenides of transition metal, such as MoS_2_, MoSe_2_, TiSe_2_, WS_2_, NbS_2_, recently are among research topics due versatile their properties. Compared with other dichalcogenides, 2D MoS_2_ architectures have attracted more attention and have been intensively investigated over the past several years. The increased interest in this semiconducting material is due to graphitic-type morphology with exciting electronic^1^,^2^, optical^3^,^4^,^5^ and catalytical^6^,^7^ properties. Besides, few-layered MoS_x_ lamellar structures in the form of crystalline^7^,^8^,^9^ or amorphous^10^,^11^ states are attributed to prospective electrocatalysts for the hydrogen evolution reaction (HER) from acidic solutions. However, 2D MoS_2_ has poor conductivity owing to semi-conductive nature and hence, poor HER efficiency. During the past few years, several methods have been reported on the formation of low charge-transfer resistant hybride^12^,^13^ and 2D defect-rich^9^ MoS_2_ materials. For example, the oxygen-incorporated MoS_2_, possessing a narrower band gap of 1.30 eV compared to that of the pristine 2H-MoS_2_ (1.75 eV) and better conductivity was proposed^14^. 2D MoO_3_ also showed excellent electrocatalytic activity for HER with the onset potential of −0.04 V *vs*. reference hydrogen electrode (RHE)^15^. It has been demonstrated that the defect-rich nanosheets possess additional exposure of active edge sites for HER generating more unsaturated sulphur atoms in the more disordered structure^9^. Besides that, inter-domain structure possesses a higher conductivity^9^.

The thickness of individual S-Mo-S layer in the pristine crystalline MoS_2_ was approximated to be equal to ≈0.63 nm^16^. In pristine crystals, the hexagonal 2H-type arrangement of atoms is more stable and dominant. As a result, rhombohedral 3R-type MoS_2_ phase transforms into 2H-type upon heating^17^. A pure 2 H phase can transform to a metastable metallic 1T phase upon Li-intercalation and exfoliation of S-Mo-S layers weakly interacting with each other by van der Walls forces^18^. In contrast to the 2 H structure, the MoS_2_ nanocrystals often exhibit a triangular morphology^19^. As reported, the triangular and hexagonal forms can be changed by varying the sulphiding and MoS_2_ formation conditions^20^. In the case of amorphous MoS_2_ materials, they usually have many defect sites and coordinately unsaturated S atoms^21^. As reported by Xie group^9^, the basal planes of MoS_2_ containing abundant defects can readily crack exposing additional active edge sites.

Except of mechanical and chemical exfoliation of pristine MoS_2_ crystals, nanocrystalline MoS_2_ can be synthesized by several methods including chemical vapour deposition^22^, thermal decomposition^23^, and hydrothermal/solvothermal treatment using different Mo and S- containing precursors^24^,^25^,^26^.

It is worth noticing, that MoS_x_-based single and few-layered materials possess a good visible light absorption^27^ and, in contrast to inert graphene, possesses versatile chemistry including various phase and compositional transformations upon annealing, storage, doping and photo-excitation. For example, it has been recently reported that edge sites of MoS_2_ monolayer are more resistant to photocatalytic degradation compared to few-layered MoS_2_ edges^27^. As a result, MoS_2_ was oxidized to MoO_x_ in an *aqua* solution with photoexcited electrons.

In this study, we report for the first time the compositional and structural transformations of few-layered MoS_2_-based nanoplatelets induced by irradiation with the continuous wave (CW) or nanosecond-pulse lasers (ns) using the wavelength of 532 nm in air demonstrating that absorbed photons can induce drastic modifications towards the increase of purity and crystallinity of MoS_2_. These processes were studied as a function of the irradiation dose of monochromatic laser light in the ambient conditions as well as the initial purity of nanoplatelet arrays formed on the Ti substrate under adapted herein hydrothermal treatment conditions. X-ray diffraction, Raman spectroscopy, X-ray photoelectron spectroscopy, and scanning electron microscopy were used to reveal the compositional and structural transformations of MoS_x_-based nanoplatelets.

## Results and Discussion

### Synthesis and characterization by SEM and XRD

**Figure 1:**
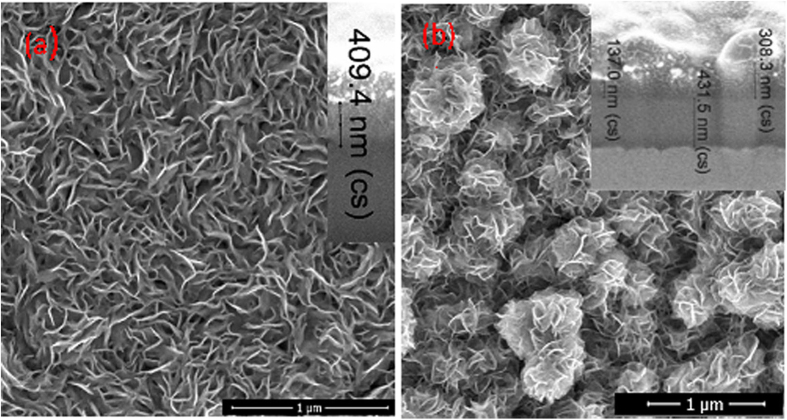
Top-side SEM images of nanoplatelet species formed on the Ti surface *via* hydrothermal treatment in the solution containing 5 mmol L^−1^ (NH_4_)_6_Mo_7_O_24_ and 90 mmol L^−1^ thiourea at 225 °C for 5 h (**a**) and 15 h (**b**). In the *Insets*, cross-sectional SEM images of the corresponding films are shown.

In this study, the synthesis of densely packed molybdenum sulphide nanoplatelet arrays on the Ti substrate was undertaken by one-pot hydrothermal synthesis way from ammonium heptamolybdate and thiourea solution under modified conditions, reported previously^28^. Briefly, 15 mL of 5 mmol L^−1^ (NH_4_)_6_Mo_7_O_24_ 4H_2_O and 90 mmol L^−1^ thiourea solution were transformed in the Teflon-lined stainless steel autoclave, 25 mL in volume, and heated at 225 °C for 5 h or 15 h using 10 °C min^−1^ ramp, then cooled down naturally to the room temperature. Upon this procedure after 5 h autoclaving, the surface of Ti sample placed vertically in the autoclave solution was covered with a black- coloured nanoplatelet array with a quite uniform thickness of ≅400 nm (Fig. 1a). More detailed SEM and HRTEM investigations revealed the formation of quite uniformly sized and densely packed nanoplatelets with the thickness of 3–6 nm at their edge sites. An increase of the synthesis time up to 15 h resulted in the formation of a film composed of nanoplatelet and nano-flowered species with approximately twice smaller width of leaflets (Fig. 1b). Besides it, significantly thicker and uneven in thickness films usually were formed.

**Figure 2:**
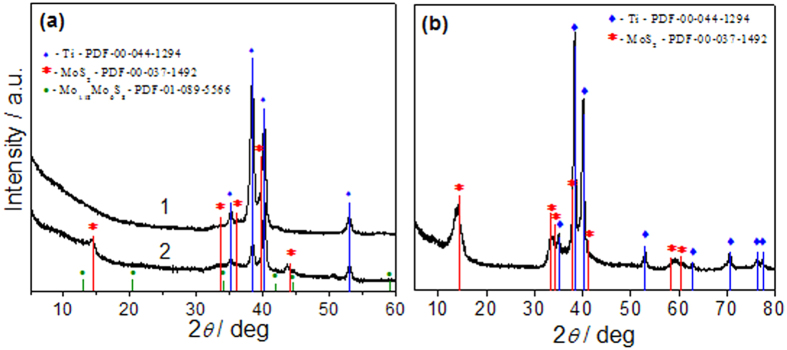
(**a**) Glancing angle XRD patterns of the specimen S5 composed of nanoplatelet film fabricated on Ti substrate by hydrothermal synthesis in the solution containing 5 mmol L^−1^ (NH_4_)_6_Mo_7_O_24_ and 90 mmol L^−1^ thiourea at 225 °C for 5 h (**a**) before (1) and after (2) green light (λ_exc_ = 532 nm) irradiation with nanosecond laser beam of average irradiation dose of 12.74 J cm^−2^. In (**b**) the same XRD plot for as-grown specimen S15 without any laser irradiation.

According to glancing angle XRD pattern, presented in Fig. 2, the crystallinity of MoS_2_ films fabricated on the Ti substrate in the adapted herein solution at 225 °C depended largely on the synthesis time. Figure 2 depicts the glancing angle XRD patterns taken from the films synthesized during 5 h (a) and 15 h (b). These specimens were labelled as S5 and S15, respectively. The array of nanoplatelets S5 possesses just low crystallinity since the observed clear diffraction peaks at 2*θ* = 35.18, 38.42, 40.18 and 53.08 deg, according to PDF card no 00-044-1294, should be ascribed to the Ti planes coming from the substrate. Increase in the synthesis time up to 15 h resulted in formation of the films excelling in good crystallinity and purity of MoS_2_ because all labelled diffraction peaks can be indexed to those of the pure rhombohedral phase of 3R-MoS_2_ with the lattice constants of a = 3.145, b = 3.145, c = 18.71 Å (Fig. 2b), which are consistent with the values of standard PDF card no. 04-004-4330.

### Laser irradiation effects probed by Raman spectroscopy

**Figure 3:**
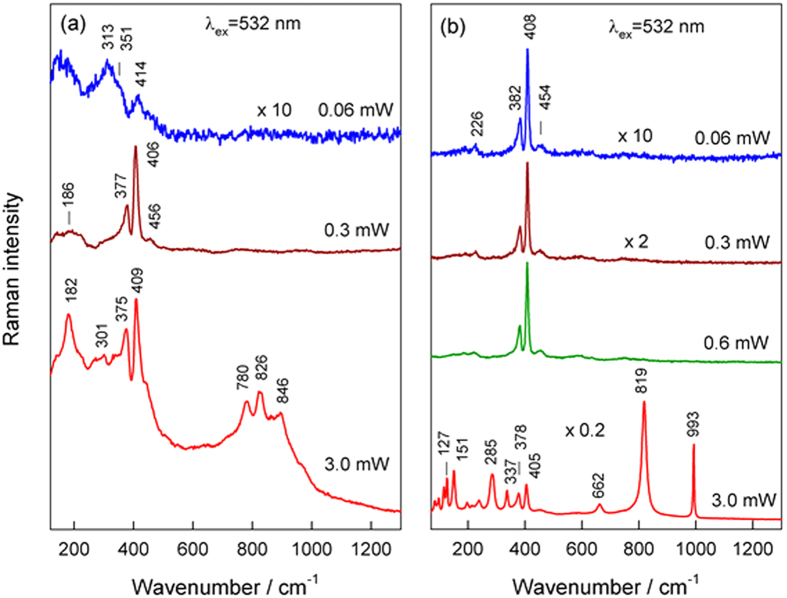
Raman spectra of the nanoplatelet arrays S5 (**a**) and S15 (**b**) specimens formed by hydrothermal treatment on the Ti surface and irradiated by the green CW laser light with indicated power.

In the next setup, the composition of nanoplatelets formed under conditions of this study was investigated by Raman spectroscopy. Raman spectroscopy is extremely sensitive to bonding strength and coordination symmetry; thus, different phases could be recognized from their distinct Raman spectra. The most studied 2H-MoS_2_ exhibits four first-order Raman bands located at 32 (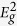), 286 (E_1g_), 383 (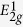), and 408 cm^−1^ (A_1g_)^29^. The intensity of 286-cm^−1^ is very low, and usually two well-resolved bands near 383 and 408 cm^−1^ are used to recognize the 2H-MoS_2_ phase^29^,^30^. Note that *A*_1g_ (408 cm^−1^) and 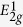
(383 cm^−1^) Raman modes are due to out-of-plane vibrations of only S atoms and opposite vibration of two S atoms with regard to Mo atom, respectively^29^,^30^,^31^. For a 1T-MoS_2_ structure, the theoretical analysis suggests the presence of two Raman active modes located at the lower frequencies, ca. 258.1 (*E*_g_) and 356.5 cm^−1^ (*A*_1g_), differing from the corresponding modes of 2H phase^31^. Because of distorted structure the 1 T′-MoS_2_ polymorph, it is expected to observe many characteristic low frequency vibrational modes located at 139.2 (A_g_), 146.3 (B_g_), 209.0 (B_g_), 216.3 (A_g_), 317.9 (A_g_), 336.0 (A_g_), and 399.8 cm^−1^ (A_g_)^32^. Considering the observed spectrum of the sample S5, taken after the green (532 nm) CW laser excitation with a low average power, namely 0.06 mW (2 kW cm^−2^), (Fig. 3a), the bands at 157 cm^−1^ and 313 cm^−1^ can be assigned to *J*_1_ and *J*_3_, modes of 1 T′-MoS_2_ structure, while the bands peaked near 351 and 414 cm^−1^ probably have some contribution from the 2H-MoS_2_ phase. Therefore, the Raman spectra of highly defected MoS_2_ specimen S5 demonstrate a low purity and amorphous structure of MoS_2_.

We found, however, that the green CW light excitation with larger power, ca 0.3 mW (10 kW cm^−2^), resulted in significant changes of the Raman response from the same sample (Fig. 3a) due to the formation of the 2H-MoS_2_ phase, clearly visible from the intense characteristic Raman bands peaked at 377 and 406 cm^−1 ^29,30. The further increase in the green light excitation power up to 3.0 mW (100 kW cm^−2^), however, resulted in the photo-degradation of MoS_2_ viewed from the appearance of Raman bands peaked at 780, 826 and 846 cm^−1^ presumably due to formation of molybdenum oxides^33^,^34^,^35^. For comparison, the same effect was studied for pure MoS_2_ nanoplatelet/nanoflowered specimen S15 synthesized in the same solution *via* increase of the synthesis time up to 15 h (Fig. 3b). In this case, no changes in the MoS_2_ structure was observed for laser irradiation power up to 0.6 mW (20 kW cm^−2^); the presence of highly crystalline 2H-MoS_2_ structure was spectroscopically evident because of the appearance of intense characteristic bands at 382 (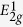), and 408 cm^−1^ (A_1g_) in the Raman spectra. Thus, the observed Raman spectra of as-grown sample S15 evidence the presence of highly crystalline and pure MoS_2_. However, at higher excitation laser power, namely 3 mW (100 kW cm^−2^), drastic changes took place in the Raman spectrum evidencing the appearance of strong peaks at 819 and 993 cm^−1^, characteristic to molybdenum oxides^33^,^34^. The strong band ascribed to the Mo–O–Mo stretching vibration serves as a useful marker for discrimination of orthorhombic (820 cm^−1^) and monoclinic (848 cm^−1^) crystal phases. The clearly defined peaks at 127, 151, 285, 337, 662, 819, and 993 cm^−1^ evidence a laser-induced formation of orthorhombic MoO_3_^33^,^34^,^35^. We hypothesized that observed laser-induced chemical transformation of MoS_2_ to α-MoO_3_ opens the possibility for simple laser writing on the MoS_2_ substrate creating the sites differing in their photo-resistance. At the same time, multiple peaks appear in a vicinity of 110 to 337 cm^−1^ instead of the typical 382 and 408 cm^−1^ bands of 2H-MoS_2_ implying on the photodecomposition of MoS_2_ and formation of non-stoichiometric molybdenum sulphides.

**Figure 4:**
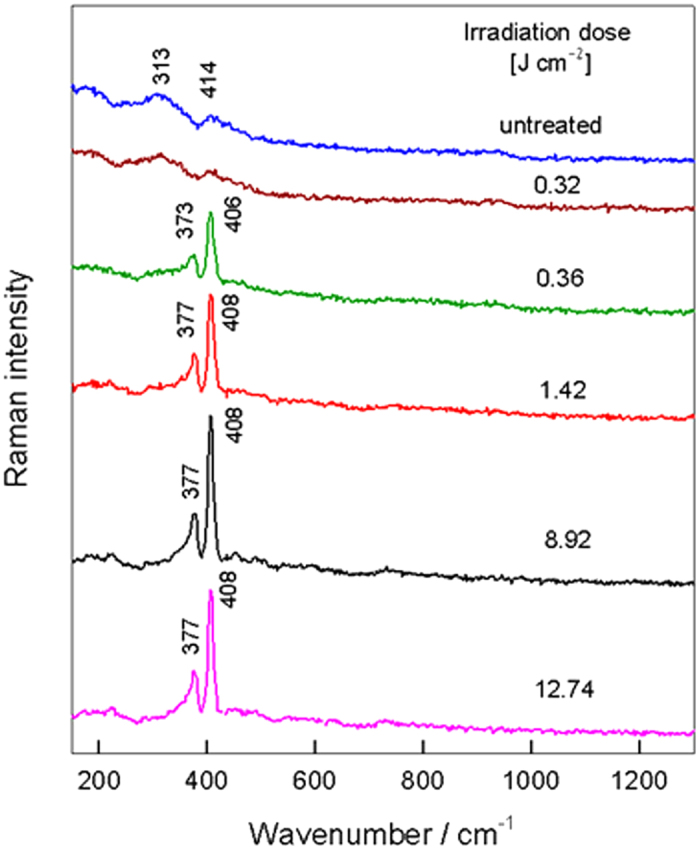
Raman spectra of the sample S5 showing the transformation of amorphous to crystalline MoS_2_ with an increasing of the nanosecond 532-nm laser irradiation dose. The excitation wavelength is 532 nm (0.06 mW).

Processing of material it is easier to conduct by using pulsed laser systems. Therefore, more detailed analysis of the effect of laser processing parameters on the irradiation-induced transformation of MoS_2_ was performed by employing the nanosecond 532-nm laser setup. Before Raman spectroscopy analysis the S5 sample was treated by ns laser irradiation; the matrix of 1 × 1 mm^2^ areas with different laser irradiation dose were fabricated. Figure 4 shows that no changes in the spectrum of amorphous MoS_2_ took place until the irradiation dose reached 0.32 J cm^−2^. Further increase in the irradiation dose resulted in typical Raman spectrum of a highly crystalline MoS_2_ with two strong bands located at 408 and 377 cm^−1^; no bands characteristic to molybdenum oxides were detected even at the highest utilised irradiation dose (12.74 J cm^−2^). This might be associated with the different irradiation effect to the material in the case of CW and pulse laser. Intensity of CW laser is quite low (3 mW corresponds to 100 kW cm^−2^) and irradiation dose is proportional to irradiation time. We can expect photo-excitation and steady elevated temperatures of the specimen. In the case of pulse nanosecond laser, intensity is in the range of MW cm^−2^, however it affects the material for a short time (during the pulse) with relaxation between pulses. Therefore, photo-excited processes should dominate in this case. Oxidation is more related to permanent heating, and it is exhibited in Raman spectra (MoO_3_ bands) only after CW laser radiation. We could expect oxidation using ns-laser but with higher pulse energy or irradiation dose.

**Figure 5:**
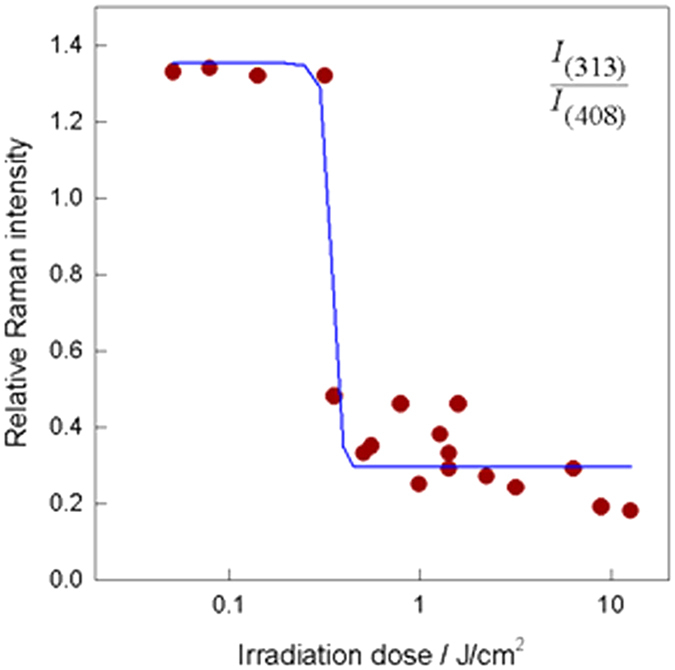
Variation of the relative intensity I_(313)_/I_(408)_ of Raman bands from specimens S5 on irradiation dose of the green nanosecond laser light (λ = 532 nm). The solid line is fitted sigmoidal form curve.

To determine quantitatively the laser irradiation dose required to induce transformation of amorphous structure to crystalline 2H-MoS_2_ phase, we have analyzed the dependence of relative intensity ratio of Raman bands I_(313)_/I_(408)_ on the irradiation dose (Fig. 5). One can see that characteristic Raman feature of the amorphous phase is located near 313 cm^−1^, while the crystalline structure shows the intense band at 408 cm^−1^ (Fig. 3). Thus, a decrease in the intensity ratio I_(313)_/I_(408)_ manifests transformation of amorphous to the crystalline phase. Experimental data were fitted by a sigmoidal form curve assuming a two-state mechanism forthe changes in Raman spectra. The transition point of the irradiation dose was found to be equal to 0.35 ± 0.007 J/cm^2^.

### Laser irradiation effects probed by XRD

XRD studies revealed (Fig. 2a (curve 2)) that illumination of nanoplatelet specimen S5 with the 532 nm nanosecond laser beam of the average irradiation dose 12.7 J cm^−2^ resulted in an increase of MoS_2_ crystallinity evident from the new diffraction peaks observed at 2*θ* = 14.40, 35.78, 39.60 and 44.16 deg (Fig. 2a). Besides that, several shoulders peaked at 2*θ* = 34.14, 44.68 and 59.26 deg, according to the PDF card no 01-089-5566, belongs to the formation of non-stoichiometric molybdenum sulphide Mo_1.18_Mo_6_S_2_.

### SEM observations of irradiated zones

**Figure 6:**
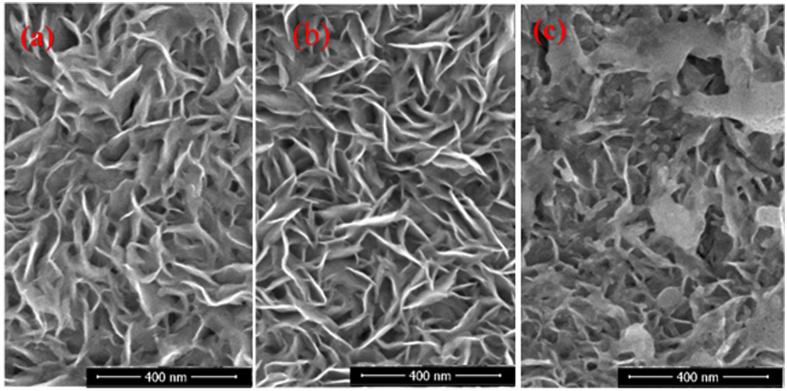
Top-side SEM view of nonstoichiometric MoS_2_ nanoplatelets formed at the Ti substrate as in Fig. 1a, before (**a**) and after (**b**,**c**) 532 nm nanosecond laser treatment with 8.92 (**b**) and 12.74 J cm^−2^ (**c**) irradiation dose.

Figure 6 depicts the top-side SEM images of nanoplatelet MoS_2_-based specimen S5 before and after the green nanosecond laser irradiation with the doses of 8.92 and 12.74 J cm^−2^. As seen, in the case of irradiation with the 8.92 J cm^−2^ dose, the design of nanoplatelets array remained the same. In the case of the 12.74 J cm^−2^ irradiation dose, obvious changes are observed. In this case, the distortion of nice nanoplatelets carpet becomes evident, although, some part of nanoplatelets edges are still visible.

### XPS Investigations of irradiated zones

**Figure 7:**
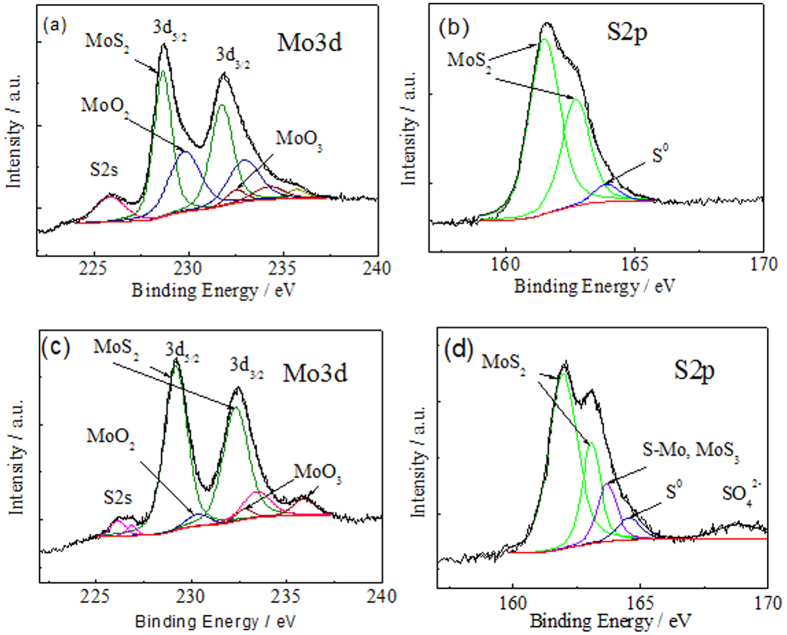
Deconvoluted XPS core level spectra of Mo 3d and S 2p elements of the specimen S5 before (**a**,**b**) and after (**c**,**d**) irradiation of the specimen S5 surface with 532 nm nanosecond laser treatment with 8.92 J cm^−2^ irradiation dose.

To determine the state and elemental composition of nanoplatelet film on the surface before and after the green light irradiation, X-ray photoelectron spectroscopy investigations were further performed. In the case of the as-grown sample S5, the XPS survey spectrum showed the presence of the Mo, S, O and C elements. The carbon peak was mainly ascribed to the adventitious hydrocarbon from the XPS spectrometer itself and was not further analysed. The high-resolution XPS spectra of Mo 3d and S 2p core level regions are shown in Fig. 7. In the case of Mo 3d spectrum of as-grown sample S5 (Fig. 7a), it can be well deconvoluted with three doublets identified according to NIST XPS database^36^ as MoS_2_ (Mo3d_5/2_ BE = 228.64 eV and 3d_3/2_ BE = 231.69 eV), MoO_2_ (Mo3d_5/2_ BE = 229.80 eV) and MoO_3_ (Mo3d_5/2_ BE = 232.94 eV). The S 2p core level XPS spectrum of the same S5 sample is fitted by three BE components peaked mainly at 161.49 eV and 162.70 eV attributable to S 2p_3/2_ and S 2p_1/2_ binding energies of the sulphur in MoS_2_. The third component with a BE = 163.89 eV and FWHM of 1.14 eV according to^36^ should be ascribed to the elemental sulphur inclusions. Judging from the BE peaks area ratio, the composition of as-grown S5 specimen nanoplatelets at the surface side should comprise of 53.20% MoS_2_, 15.5% MoO_3_, 23.7% MoO_2_ and 7.6% S^0^, indicating the dominant amount of MoS_2_.

The irradiation of the same S5 sample with the with 532 nm nanosecond laser with 8.92 J cm^−2^ irradiation dose resulted in obvious changes of the Mo 3d (Fig. 7c) and S 2p (Fig. 7d) core level spectra counterparts. First of all, the content of MoO_2_, MoO_3_ and S^0^ inclusions decreased down to 12.2%, 6.15% and 3.9%, whereas the content of MoS_2_ increased up to 77.7% correlating well with the Raman spectra changes. Secondly, from the deconvolution of the S 2p core level spectrum of the irradiated S5 specimen (Fig. 7d) two additional BE peaks, in addition to those attributable to MoS_2_ (BE = 161.96 eV and BE = 163.96 eV) and S^0^ (BE = 164.54 eV) (FWHM 0.90 eV) and BE = 168.84 eV (FWHM 1.07 eV) appeared which, according to^36^,^37^, should be ascribed to the formation of Mo(VI) sulphides and SO_4_^−2^ group, respectively.

## Conclusions

This work reported the extraordinary effect induced by the green (532 nm) CW and ns-laser light induced of the compositional and phase transformations of 2D MoS_x_-based nanoplatelets containing some amount of MoO_2_ and MoO_3_ and elemental sulphur towards significantly more crystalline and pure MoS_2_ without obvious morphological changes. Consequently, this study established a novel method to increase the purity of MoS_2_ nanoplatelet arrays formed by the one-pot hydrothermal synthesis approach without additional annealing and sulphidation procedures. The effect was seen for *visible* green (*λ*_exc_ = 532 nm) laser light irradiation when the suitable irradiation dose was applied. An apparent decrease in the intensity ratio of I_(313)_/I_(408)_ manifested transformation of amorphous to crystalline MoS_2_ phase. From the XPS observation, these findings seem to be ascribed to the formation of molybdenum disulphide by reaction of elemental sulphur in the oxygen vacancy sites formed throughout the decomposition of MoO_2_, as thermodynamically less stable, upon absorption of incoming photons energy.

## Methods

### Synthesis

Pure MoS_2_ nano-flowered species were synthesized as follows. At first, 15 mL of aqueous solution containing 15 mmol L^−1^ ammonium heptamolybdate, (NH_4_)_6_Mo_7_O_24_ 4H_2_O, and 270 mmol L^−1^ thiourea was poured in a Teflon lined stainless steel autoclave in the volume of 25 mL. The synthesis was conducted at 225 °C for 15 h using 10 °C min^−1^ ramp. The synthesized products were collected by centrifugation, rinsed carefully with pure water and dried. To obtain the carpet of densely packed MoS_2_ nanoplatelets on the Ti substrate, the sample was inserted into a same but three times less concentrated solution and kept at optimized 225 °C for 5 h or 20 h using 10 °C min^−1^ ramp. The black coloured sample was carefully rinsed and dried naturally in the air.

### Structure Characterization

For TEM sample preparation, a drop of the solution with the synthesized product was placed onto a 300 mesh copper grid covered with a carbon film. After removing an excess of the solution with an adsorbent paper, the sample was dried and investigated using a high-resolution transmission electron microscope HRTEM model FEI TECNAI F20 at an accelerating voltage of 120 keV. The morphology and elemental composition of the products obtained were investigated using a scanning electron microscope (FEI Quanta 200 F) and a Cross Beam Workstation Auriga equipped with a field emission gun and EDX spectrometer. The glancing angle XRD spectra were collected on a D8 diffractometer (Bruker AXS, Germany), equipped with a Göbel mirror as a primary beam monochromator for CuK_α_ radiation.

### X-ray photo electron spectroscopy

XPS measurements were carried out using an ESCALAB MKII spectrometer equipped with a new XR4 twin anode. The non-monochromatised MgK_α_ X-ray source was operated at *hν* = 1253.6 eV with 300 W power (20 mA/15 kV) and the pressure in the analysis chamber was lower than 5 × 10^−7^ Pa during spectral acquisition. The spectra were acquired with an electron analyser pass energy of 20 eV for narrow scans and resolution of 0.05 eV and with a pass energy of 100 eV for survey spectra. All spectra were recorded at a 90° take-off angle and calibrated using the C 1 s peak at 284.6 eV. The spectra calibration, processing, and fitting routines were done using the Avantage software (5.918) provided by Thermo VG Scientific. Core level peaks of Mo 3d, O 1 s, Pt 4 f and S 2p were analysed using a nonlinear Shirley-type background, and the calculation of the elemental composition was performed on the basis of Scofield’s relative sensitivity factors.

### Laser-Induced Selective Phase Transition Procedure (LISPTP)

**Figure 8:**
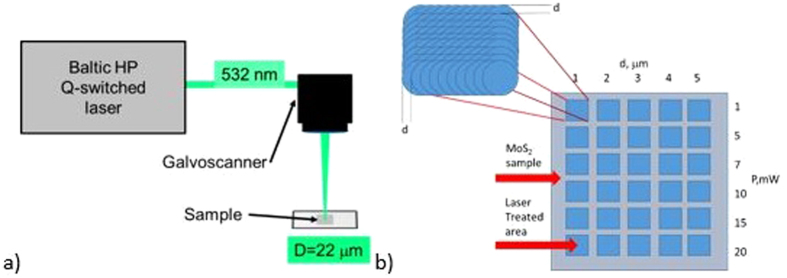
Scheme of MoS_2_ laser treatment (**a**) and the sample pattern (**b**). Laser processing setup: Baltic HP Q-switched nanosecond laser from Ekspla, galvoscanner Hurryscan (532 nm).

The LISPTP procedure was performed in order to investigate the influence of a green (λ = 532 nm) nanosecond laser irradiation on the structural and compositional transformations of the films composed of MoS_2_-based nanoplatelets. Experimental setup (Fig. 8) consisted of the nanosecond laser Baltic HP (irradiation wavelength – 532 nm, pulse duration - 10 ns, pulse repetition rate - 100 kHz) beam expander and galvoscanner with an f -theta lens focusing objective (focal distance of 80 mm for 532 nm). Areas of 1 × 1 mm^2^ were scanned in the line stacks patterns with the laser beam. Distances between adjacent lines and scanning speed (100–500 mm/s) were adjusted to ensure the same pulse overlap in both directions. The distance between adjacent laser pulses *d* - was varied between 1 and 5 μm. The beam diameter measured on stainless steel according to described methodology^38^ at the focus was 22 μm. The irradiation dose was evaluated by multiplying laser fluence by a number of pulses per focused beam spot. The irradiation dose was varied between 0.05 to 12.74 J cm^−2^.

### Raman

The 532-nm excited Raman spectra were recorded using inVia (Renishaw) spectrometer equipped with thermoelectrically cooled (−70 °C) CCD camera, 1800 lines/mm grating, and a microscope. The 532 nm beam of the CW solid state laser was used as an excitation source. Position of Raman spectra bands on the wavenumber axis was calibrated by the silicon peak at 520.7 nm. The 50x/0.75 NA objective was used during the measurements. The beam was focused to a 2 μm diameter spot size on the sample surface. The integration time was 100 s. Spectra are not smoothed. Parameters of the bands were estimated by fitting the experimental spectra with Gaussian-Lorentzian shape components using GRAMS/A1 8.0 (Thermo Scientific) software.

## Additional Information

**How to cite this article**: Jagminas, A. *et al*. Laser Light Induced Transformation of Molybdenum Disulphide-Based Nanoplatelet Arrays. *Sci. Rep.* **6**, 37514; doi: 10.1038/srep37514 (2016).

**Publisher’s note:** Springer Nature remains neutral with regard to jurisdictional claims in published maps and institutional affiliations.
